# DARAL: A Dynamic and Adaptive Routing Algorithm for Wireless Sensor Networks

**DOI:** 10.3390/s16070960

**Published:** 2016-06-24

**Authors:** Francisco José Estévez, Peter Glösekötter, Jesús González

**Affiliations:** 1 Department of Electrical Engineering and Computer Science, University of Applied Sciences of Münster, Stegerwaldstr. 39, D-48565 Steinfurt, Germany; gloesek@ieee.org; 2 Department of Computer Architecture and Technology, University of Granada, Periodista Daniel Saucedo Aranda, S/N, 18071 Granada, Spain; jesusgonzalez@ugr.es

**Keywords:** network routing algorithm, WSN, Smart City, RPL, AODV, IEEE 802.15.4

## Abstract

The evolution of Smart City projects is pushing researchers and companies to develop more efficient embedded hardware and also more efficient communication technologies. These communication technologies are the focus of this work, presenting a new routing algorithm based on dynamically-allocated sub-networks and node roles. Among these features, our algorithm presents a fast set-up time, a reduced overhead and a hierarchical organization, which allows for the application of complex management techniques. This work presents a routing algorithm based on a dynamically-allocated hierarchical clustering, which uses the link quality indicator as a reference parameter, maximizing the network coverage and minimizing the control message overhead and the convergence time. The present work based its test scenario and analysis in the density measure, considered as a node degree. The routing algorithm is compared with some of the most well known routing algorithms for different scenario densities.

## 1. Introduction

Smart City is a concept that is rising in the last years. It is not only an academic topic, but also a trending topic for political and public organizations. Its relevance is pushing research groups around the world to analyse and study how to develop and to connect it with citizenship. Recent projects and studies show the relevance of this topic [1,2,3], dealing with how to instantiate it.

Technology is moving forward very quickly and what some years ago was expensive and unsustainable is feasible today. Thus, embedded electronics are a very interesting option today for the development of a Smart City infrastructure. It presents different possibilities like 8-, 16-, 32- or 64-bit architectures, low-power systems, cable- or wireless-communications, etc. It is possible to build up a specific system with relative ease and a contained cost. Some examples are systems like Raspberry Pi Zero [4] or Arduino [5]. With the expansion of affordable embedded systems such as the cited ones, another necessity as to the communication appears. As long as the systems are small in size, these communications need to be wireless, also allowing the ability to reach places difficult to access and expand the available applications.

Technology is already capable of supporting projects like Smart City, but there are still other issues to be solved such as the number of nodes a Smart City needs. Well, that is an uncertain question; it depends on the service offer and the government. However, considering large-size scenarios such as Smart Cities or Internet of Things, there is always a common actor, the infrastructure, which is usually based on a combination of fixed and mobile devices. The infrastructure must provide the support for many other services, spreading out the devices as much as possible in order to completely cover a certain scenario. A good approach to answer the question previously formulated, is the work of Calderoni et al. [1] or even the work of Sánchez et al. [6], where they presented precise data about the ideal Smart City node distribution. Using the data published in these works as reference, the conclusion is that the total number of nodes of each particular scenario depends on its size, and thus the node density appears as a fundamental parameter in Smart City deployments. The node density of a large infrastructure like a Smart City can be defined as medium or low, depending on the available services in the scenario. The definition of *node density* can be found in the work of Kermajani et al. [7], which defines the high, medium and low density as 15, 10 and 5, respectively.

The wireless communication field is wide but there are some well-known standards that regulate it and mark the development. Some of these standards are the ones from the Institute of Electrical and Electronics Engineers (IEEE) *IEEE 802.11* [8], *IEEE 802.15.1* [9], *IEEE 802.15.3* [10] and *IEEE 802.15.4* [11]. These technologies are a major area of research, mainly *IEEE 802.11* and *IEEE 802.15.1*, but considering the scenario and topic of this work, *IEEE 802.15.4* is the most suitable standard because it is oriented towards wireless sensor networks (WSN) and low-rate communications. This standard is well-known in industrial sectors or areas like home automation, so it is also logical to extend its application to the Smart City. As *IEEE 802.15.4* regulates only the access to the medium by definition of the physical (PHY) and medium access (MAC) layers, there exist different routing algorithms, due to the lack of an advanced routing method in the standard. Star and peer-to-peer topologies are standard-defined but cluster-tree and mesh (also defined by *IEEE 802.15.5* [12]) are extended by algorithms in upper layers. These routing algorithms are needed for complex scenarios, and indeed the Smart Cities are one of the most complex possible scenarios. Moreover, other features more than topology are required, and routing algorithms are fully qualified to provide them. Reliability, granted Quality of Service (QoS), energy efficiency, duty cycle control, synchronization, etc., are some necessities for modern wireless communications that can only be covered by advanced routing algorithms.

*IPv6 Routing Protocol for Low-Power and Lossy Networks (RPL-6LoWPAN)* [13] or *Ad-Hoc On demand Distance Vector (AODV)* [14] are some of these well-known *IEEE 802.15.4*- based routing algorithms. Each one possesses some interesting features, but they also present disadvantages when they are exposed to a scenario like the Smart City. The present work focuses on the analysis of set-up time and protocol overhead due to the necessity of a fast configuration and a fast self-healing, due also to energy and throughput requirements (a lower overhead requires a lower consumption and allows a better communication channel use).

This article addresses the problems of convergence time [7] through different scenarios, together with the control overhead at network formation phase, based on a comparison between other well-known routing algorithms. However, the current routing algorithms for WSN do not usually take into account these key features for Smart City scenarios, and they leave space for new proposals as the one presented here.

*DARAL* proposes a routing algorithm based on a dynamically-allocated hierarchical clustering, which uses the quality of the links as a reference parameter, minimizing the control message overhead, convergence time and energy consumption during the set-up phase. To achieve energy-efficiency, the nodes minimize their active time transferring the routing tasks to some dynamically-selected nodes. Additionally, *DARAL* organizes the nodes in virtual sub-networks, containing the traffic by zones and also incrementing the efficiency in terms of convergence time.

The rest of the paper is organized as follows: Section 2 provides an overview of the most relevant related work about routing algorithms for WSN. Section 3 describes *DARAL*, the routing algorithm proposal designed for a fast set-up and low-overhead in low-/medium-density scenarios. Section 4 presents the simulations and results obtained with our algorithm. Finally, the most relevant conclusions of this paper are explained in Section 5.

## 2. Routing Algorithms for WSN

Routing algorithms are an important part in Smart City and Internet of Things (IoT) projects, due to the critical function they play. Although there are different routing methodologies like multicast [15], mesh [16] or graph-based [17], routing algorithms based on clustering [18,19,20] are designed to improve different parameters such as QoS [21], network lifetime [22], energy consumption [23], traffic reduction or range maximization [24]. Thus, our proposal, DARAL, is based on clustering techniques. Within clustering-based routing algorithms, there are mainly hierarchical cluster tree algorithms, but other alternatives exist like the clustering mesh-like alternative proposed by Wang in [18] or the spanning tree proposed by Saravanan et al. in [25].

Usually, the implementation of a hierarchical clustering scheme is based on the definition of two different roles or functionalities for the nodes of the network, the cluster-heads and non-heads nodes. A cluster is formed by a cluster-head and a set of non-head nodes, where the nodes of the cluster communicate between them (sensor-to-sensor) and mainly with their cluster-head, which also leads the inter-cluster communications.

As the role selection mechanism is a fundamental aspect in hierarchical cluster-based routing algorithms, there exist different cluster-head election schemes that consider a wide range of parameters such as location, residual energy or Link Quality Indicator (LQI). For example, Jiasong et al. [26] described an adaptive routing optimization based on the energy balancing algorithm for hierarchical networks in ZigBee, where they limited the number of hops depending on the battery available, limiting the range of a certain node as well. Another possibility for the cluster-head election is the one proposed by the MultihopLQI routing algorithm [27], where a tree of multiple hops is dynamically built for routing tasks by the analysis of the impact of an LQI threshold in the routing formation, considering *MinLQI* and *MaxLQI* values. However, this cluster-head election scheme does not take into account some important parameters such as QoS, convergence time or control overhead, focusing instead on the analysis of path length and network lifetime. This is a common pitfall in most of the common cluster-head election schemes. As long as they need to analyse different parameters to produce a measurable result, they suffer from two major drawbacks: an increment of the convergence time and also a message overhead, making them unsuitable for Smart City or IoT projects.

There is also a wide diversity in hierarchical cluster-based routing algorithms, mainly due to different scenario characteristics. Thus, each routing algorithm usually focuses on a specific set of parameters related to a concrete scenario, looking for their improvement. For example, Chang et al. [28] explored the maximum lifetime of the routing in WSN through the reformulation of the energy efficiency problem. They improved the total energy efficiency of the system using the network lifetime increment instead of minimizing the total energy consumed to reach the destination. Another approach is presented by Huang et al. in [29], where the problem of the coverage for WSN is analysed, assuming that all the nodes are under a certain coverage grade. However, focusing on Smart City/IoT applications, and as Machado et al. commented in their article [30], routing solutions for these scenarios should consider different traffic patterns, such as one-to-many, many-to-one and many-to-many, due to the dynamical nature of Smart City/IoT applications. This requirement has favoured that most of the Smart City/IoT proposals are based on two well-known routing algorithms: AODV and RPL, mainly because both present low-complexity and good performance in these scenarios.

*AODV* is a routing algorithm that discovers the routes using Route Request (RREQ) and Route Reply (RREP) messages, ensuring that there are no loops and, at the same time, trying to find the shortest possible route. A certain origin node broadcasts a RREQ to their neighbour nodes, looking for a certain destination node. These neighbours look in their routing tables for the destination node. If there is not any match, these neighbours also broadcast the original RREQ to other nodes, and the process continues until a node finds the destination node in its routing table. However, if there is a match, the node with the match resends the original RREQ to the destination node and once the destination node processes the RREQ, it answers with a RREP that is routed towards the origin node, setting the route and closing the route discovery process. As this routing method is on-demand fired, and it is based on a minimal number of hops, it does not ensure neither energy-efficiency, nor convergence time or control overhead efficiency. Moreover, *AODV* only stores one route for a destination node, resulting in an additional use of resources if there is any problem with that route, due to the necessity of discovering a new one. The lack of a mechanism to ensure energy, convergence time efficiency and a minimal control overhead results in a waste of resources for constrained scenarios with low-/medium-densities, due to the instability of links and routes.

On the other hand, *RPL* routes are based on Destination Oriented Directed Acyclic Graphs (DODAGs). A *DODAG* is a graph based on nodes and links forming the path to the network root, which is basically the sink or network coordinator. Thus, *RPL* has been designed and optimized for the transmission of data from sensor nodes towards the root node, following a many-to-one scheme. For the construction and maintenance of a *DODAG*, *RPL* nodes locally multicast *DODAG* Information Object (DIO) messages pseudo-periodically, which contain information that allows a node to discover an existing *DODAG*, jointly with its configuration parameters. Once a node receives a DIO message from a neighbour, it will be able to join an existing *DODAG*. However, if a node is trying to connect the network and does not receive any DIO message, it can send a *DODAG* Information Solicitation (DIS) message to request the immediate transmission of DIO messages. Once the DIS message is sent, additional DIS messages may be sent until a DIO message is received in response. However, if a DIO message is not received after a certain time, the node may decide to become the root of a new *DODAG*. *RPL* also supports downwards routes by using Destination Advertisement Object (DAO) messages, which are generated and sent upwards by non-root nodes to announce themselves as possible destination nodes. The lack of energy and convergence time efficiency, jointly with the control overhead, results, as in the *AODV* case, in a waste of resources. This problem was analysed by Kermajani et al. in [7], where an optimized *RPL* *DODAG*-route forming method (O-RPL) was proposed to reduce the convergence time for dense scenarios [7]. This also shows an interesting test-bed to analyse both the convergence time and control overhead in terms of node degree (density).

As described above, existing routing algorithms present some drawbacks related to the lack of an integrated QoS, an efficient convergence time and a minimal control overhead in the network formation stage for Smart City/IoT scenarios, considering these scenarios as low-/medium-density environments. With this goal in mind, we propose a new routing algorithm based on the LQI, which is hierarchically organized in sub-networks, that improves the convergence time and the message control overhead for Smart City/IoT scenarios with a small-/medium-density where the coverage is a critical parameter.

## 3. Dynamic and Adaptive Routing Algorithm (DARAL)

The novelty of this paper is the development of a centralized non-beaconing routing algorithm based on a dynamical clustering, which is based on the link quality between nodes. *DARAL* is a routing algorithm, that works on top of the *IEEE 802.15.4* standard, based on the idea of DARP [31]. DARP is the first approach to the DARAL proposal, and it has been developed to efficiently cluster nodes in sub-networks. Based on these ideas, DARAL has been designed for low-/medium-density scenarios, like Smart Cities, focusing on infrastructure support. The clusters are configured like virtual sub-networks, as in DARP, but they can now work autonomously and in parallel, due to the parallel execution of the algorithm in every node. Therefore, every node, except for the network coordinator, make use of the *Dynamical Role Selection Process* or *DRSP*, an algorithm that is also part of *DARAL* and it is used as cluster creation and link selection mechanism.

**Figure 1:**
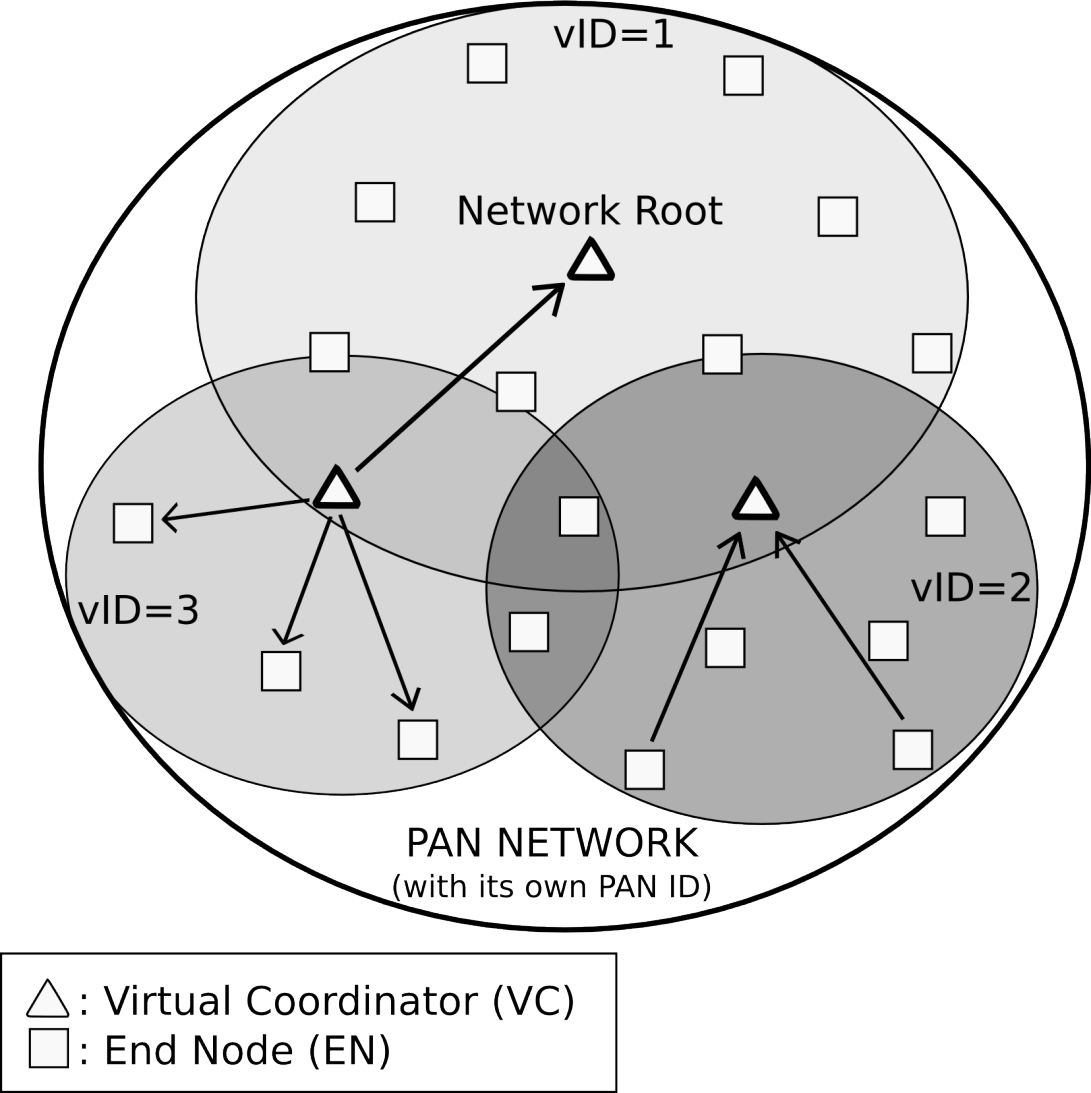
*DARAL* basic concepts.

Before entering in a fine grain description, it is necessary to define some general concepts about *DARAL*, like the sub-network concept and the different types of roles. The sub-network concept comes from the clustering techniques and the idea of Smart City organization. In Smart City approaches, nodes usually communicate between them in the neighbourhood. Thus, if clustering is applied, the traffic can be contained in a determined area, allowing the reduction of interferences with other nodes and also minimizing the necessity of looking for a node among the complete network topology. In order to improve the clustering concept, the virtual sub-network concept extends this idea, applying it in routing tasks. Each virtual sub-network is identified with a virtual identification (vID), which is used to route messages among the network tree. Figure 1 shows a global Personal Area Network (PAN) with its own identification, but internally sub-divided in three different sub-networks. The first one is where the network root is located, while the other two group different nodes.

Another general concept about *DARAL* is the node role, considering that every node should be similar in terms of hardware. *DARAL* runs DRSP at the start-up of every node, which allows selecting the role to be played by a node in the network. Figure 1 shows the two available roles in *DARAL*, end node (EN) and virtual coordinator (VC). ENs are nodes that only communicate with the VC of its sub-network. VCs have the same functionality as ENs but also manage virtual sub-network and store routing tables.

### 3.1. Node States

**Figure 2:**
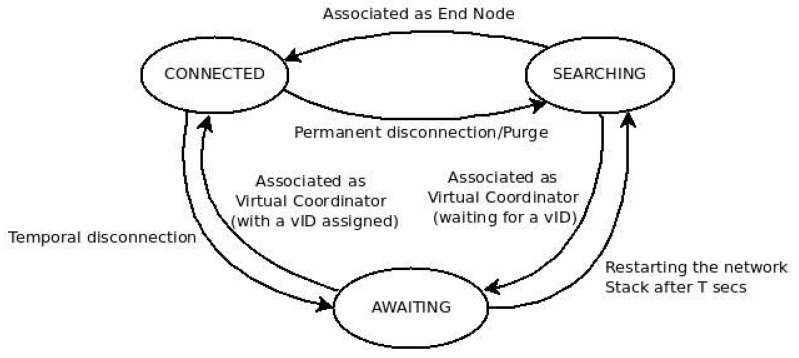
*DARAL* state machine.

As Figure 2 shows, each node of the net can be in one of the three node states defined in *DARAL*: *SEARCHING*, *AWAITING* and *CONNECTED*. In either *AWAITING* and *CONNECTED* states, a node can send and receive data messages, but a VC cannot route packets as long as it is not in the *CONNECTED* state.

An EN only changes between two states, *SEARCHING* and *CONNECTED*, although a VC transits the three states, but only with connection purposes. Both VCs and ENs start in the *SEARCHING* state, and as long as they finish the execution of *DRSP*, the EN changes to *CONNECTED* and the VC changes to *AWAITING*. In the *AWAITING* state, a VC requests a new vID for its sub-network, and once it is received, it updates its information and changes to *CONNECTED*, allowing other nodes to connect with it.

**Figure 3:**
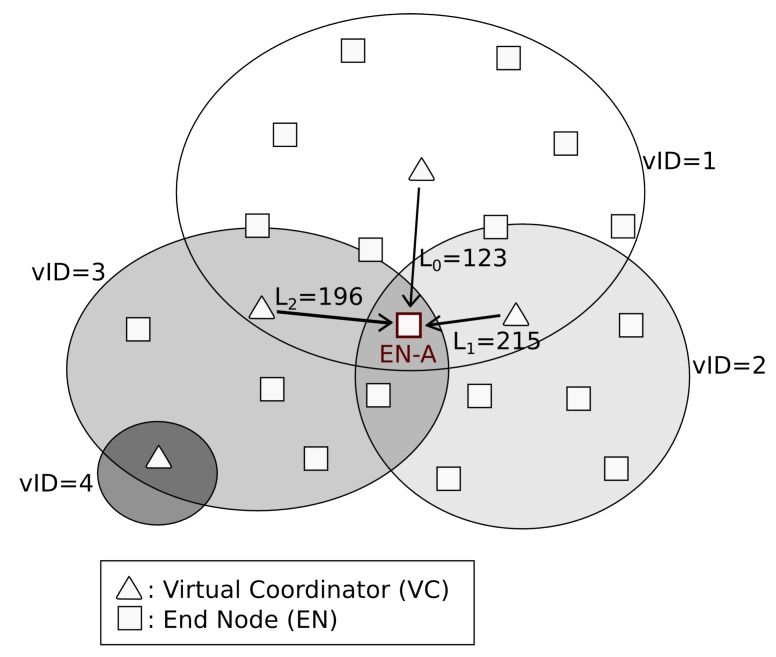
Example of node collecting LQIs for the link selection and role adoption process.

**Figure 4:**
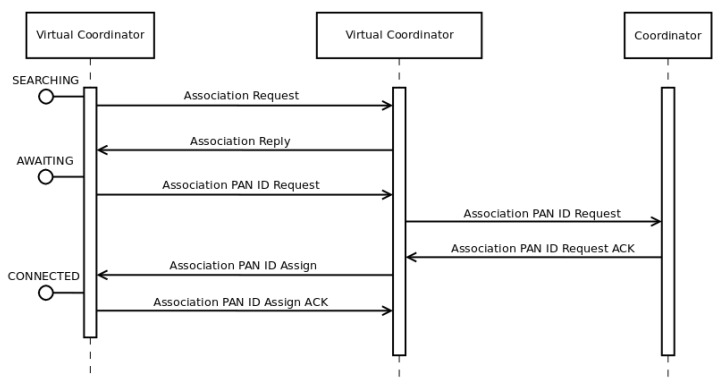
Message flow in a VC association operation.

**Figure 5:**
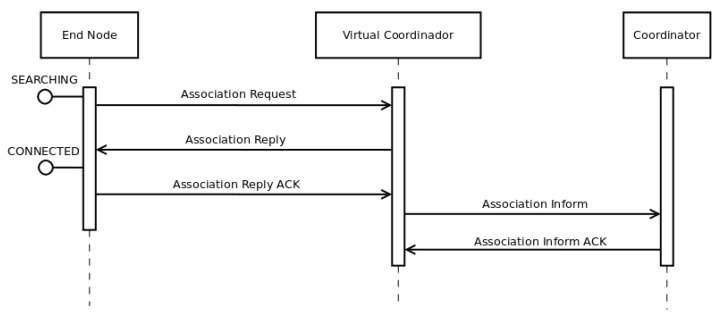
Message flow in an EN association operation.

Figure 3 shows the first process in *DARAL*, where a node *EN-A*, which is in *SEARCHING* state, tries to connect to the network. Figure 4 and Figure 5 show the message flow to carry it out. First of all, *EN-A* broadcasts an *ASSOCIATION_REQ* message. In Figure 3, the VCs from sub-networks 1 (VC-1), 2 (VC-2) and 3 (VC-3) are within its communication range, so they are receiving the *ASSOCIATION_REQ* message. After checking the message, they proceed to answer with an *ASSOCIATION_REP* message. *EN-A* receives those *ASSOCIATION_REP* from VC-1, VC-2 and VC-3 and fires the *DRSP* algorithm (Section 3.2), in order to choose the best sub-network to join, in terms of link quality indicator (LQI). Once a sub-network is chosen and *EN-A* selects its role, *EN-A* changes its state to *AWAITING* or *CONNECTED* and sends a message to the corresponding VC, VC-2 in Figure 3, and from then on it belongs to that sub-network, i.e., sub-network 2 for this example.

### 3.2. Cluster Creation Using the Dynamic Role Selection Process (DRSP)

**Figure 6:**
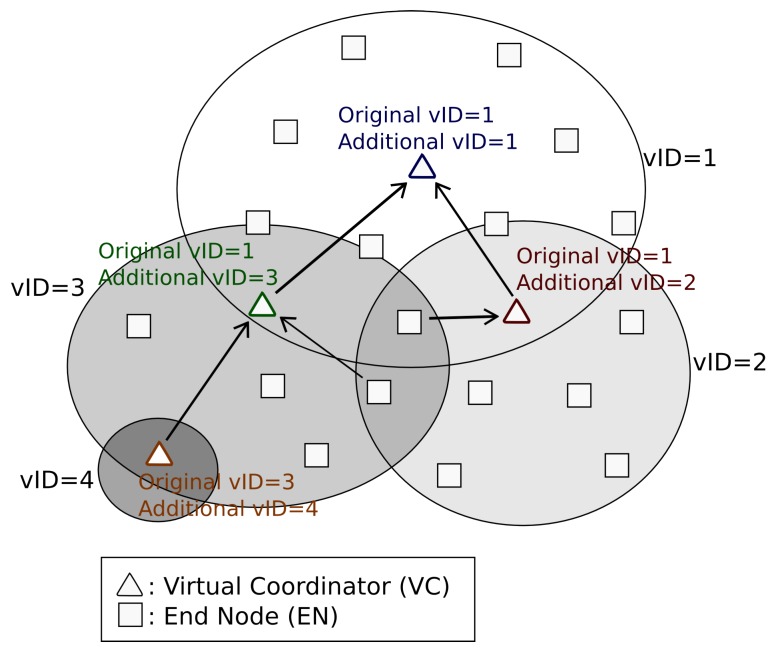
Example of virtual sub-networks’ organization.

As Figure 3 shows, a node selects the best link to connect with and, once it is chosen, the node evaluates the LQI to adopt one of two possible roles in the network, EN or VC. EN is basically limited to receiving and sending messages, reducing as much as possible its active time, thus saving energy. On the other hand, VCs are the key elements in *DARAL*, being used to widen the network coverage range and to route messages among sub-networks. VCs play both roles at the same time, which can be seen in Figure 6, as ENs for the network they belong to and as VC to the sub-network that they own. In Figure 6, every VC shows their two vIDs, the *original vID* from the network where they belong, and the *additional vID* from the sub-network that they manage.

*DRSP* is based on two user-defined parameters: $TH_{baselevel}$ and $TH_{role}$. $TH_{baselevel}$ is basically a threshold granting a minimum QoS for the link with the VC. Any link with a LQI below $TH_{baselevel}$ is not established. On the other hand, $TH_{role}$ defines the threshold for the role. If the LQI is above $TH_{role}$ a node configures itself as EN, due to the good QoS of the link. However, if the LQI is below $TH_{role}$ (and above $TH_{baselevel}$), a node adopts the role of VC, considering that from that point in advance the QoS will worsen even more. *DARAL* is based on the concept that a high LQI (above $TH_{role}$) means a better QoS and a nearest position, allowing grouping nodes and a traffic concentration, hence the adoption of EN role. A worse LQI (below $TH_{role}$) represents a point where the QoS is not good enough, and if the distance continues increasing, that QoS will worsen. To reduce the impact of the deterioration in the QoS, a VC acts as a concentrator, which allows for expanding the network coverage.

**Figure 7:**
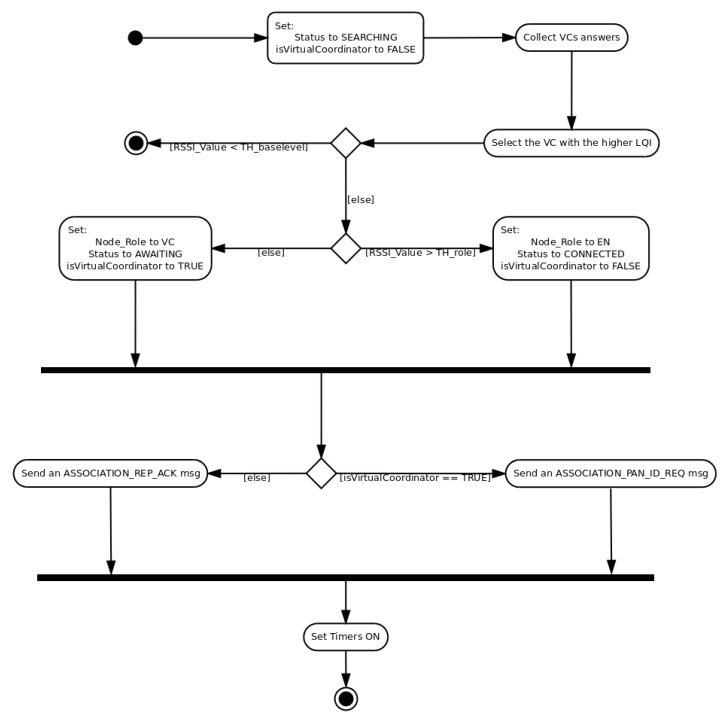
Dynamical role selection process (DRSP).

Figure 7 shows a UML description of *DRSP*, detailing how it is carried out. First of all, it is necessary to set some internal parameters to their default values, and then collect some VC answers. The third step is the selection of the answer with the higher LQI, and then the $TH_{baselevel}$ is evaluated. If the LQI is below $TH_{baselevel}$, the process finishes. Otherwise, if it is above $TH_{baselevel}$, then it continues evaluating the $TH_{role}$ value. If the LQI is above the $TH_{role}$, then the role is set to EN, the node status changes to *CONNECTED*, and the node is marked as non-VC. If the LQI is below $TH_{role}$ (and above $TH_{baselevel}$), then the role is set as VC, the status is set to *AWAITING*, and the node is marked as VC. The *AWAITING* status means that a VC is connected and can receive messages, but, at the moment, cannot route packets. This status changes to connected when that VC receives a vID for its new sub-network.

Finally, the algorithm evaluates the node role adopted to send an *ASSOCIATION_PAN_ID_REQ*, if the role is VC, or an *ASSOCIATION_REP_ACK*, if the role is an EN. At the end, different timers, like packet delivery time or connection status time, are set.

Each node in the network, except for the coordinator, executes *DRSP*, dynamically creating the network hierarchy. The nodes analyse the potential connections and choose the best one. Once the node is established, the connection continues. If the node is an EN, it does nothing more, and only communicates with its VC when it is needed. However, if the node is a VC, the node asks for a vID due to the creation of a new sub-network. Once the new vID is set, the sub-network is also created and the node acts as a potential father for other nodes, continuing the process until no more nodes ask for connection.

### 3.3. Sub-Network Concept

*DARAL* routing is based on the use of sub-networks. Every sub-network possesses a sub-network ID (vID), which unequivocally identifies the sub-network in the whole network. Each cluster or sub-network groups a certain number of nodes, due to the number of nodes user-limited by the parameter $L_{nodes}$, hence the load balance of *DARAL*. If the limit $L_{nodes}$ is reached by a VC, that VC does not send any *ASSOCIATION_REP* anymore, at least, as long as the number of connected nodes remains unchanged.

VCs manage the sub-networks, possessing two different routing tables, one for the local nodes in the sub-network and another for routing tasks. VCs know only the routes below their position, for example in Figure 6, the VC from sub-network 3, knows the nodes in its sub-network and the nodes in sub-network 4. In Figure 6, the network root, (VC in sub-network 1) also knows the complete routing table. The particularity of *DARAL* is that a message being routed between two different sub-networks is not completely processed. A VC in the route only needs to analyse the *Destination Virtual Network ID*, and look for it in the routing table, to know the next step in the route. Only messages that have already reached their destination sub-network are completely processed. Then, the VC of that sub-network looks for the destination address in the local nodes table.

Sub-network IDs (vIDs) are only generated by the network root. Once a vID is generated, all the VCs in the route update their routing tables, adding a new entry to them. On the other hand, if a new EN connects to a sub-network, the VC of that sub-network informs every node above him, sending an *ASSOCIATION_INFORM* message. Every VC that receives an *ASSOCIATION_INFORM* updates its own routing table.

**Figure 8:**
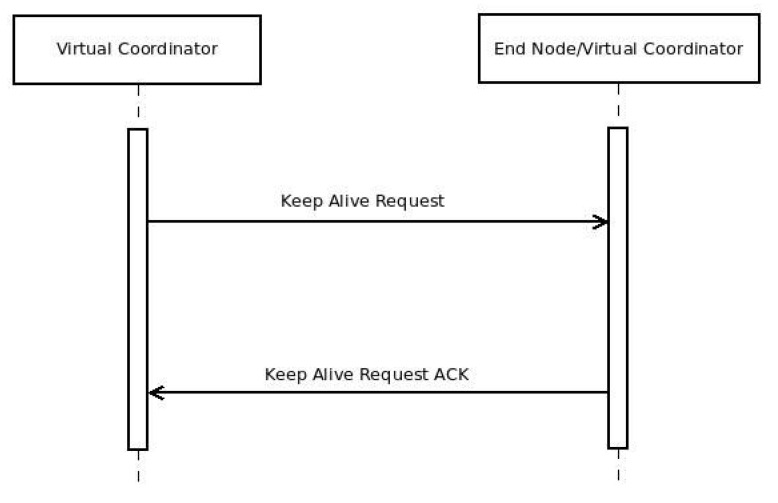
Message flow in a successful control check or keep alive operation.

**Figure 9:**
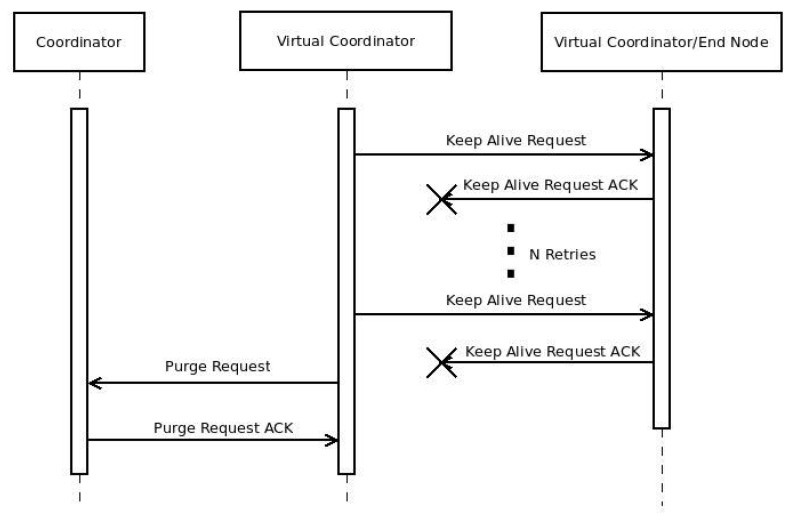
Message flow in a control check or keep alive operation with errors.

Due to this routing mechanism, it is necessary to check periodically the status of the different sub-networks, as well as the connectivity of the ENs. Figure 8 shows this process, which is fired by the user-defined timer $T_{alive}$. When $T_{alive}$ is expired, a VC sends a *KEEP_ALIVE_REQ* message to its sub-network, checking the connectivity of every node. If a node does not respond like in Figure 9, another user-defined timer $T_{down}$ is fired. When $T_{down}$ expires, a purging request is generated by the VC, asking the VCs above him to remove that node from the routing tables.

If the connectivity problem is detected by ENs and not by a VC, those ENs wait until the user-defined timer $T_{reconnect}$ is expired. If the timer expires and there is no answer from the VC, those ENs restart and begin with *DARAL* and the *DRSP*, doubling the value of $T_{reconnect}$. If the time expires once more, $T_{reconnect}$ returns to its normal value, following a scheme *t*–2t–...–*t*–2t. After restarting, the nodes reconnect the network and the routing tables are updated with the new information. If the keep alive process starts during the disconnection, a purge process is fired.

### 3.4. Sub-Network Routing

The routing between different sub-networks is strictly necessary for the right functioning of *DARAL*. Thus, and as can be seen in Appendix A, there exist a specific field called *routing type* in the protocol header, which defines the three different routing methods in *DARAL*:

- Gateway: It represents a message going up in the network tree, using the link with the upper VC. This routing type is based on the sub-network identification or vID to route packets.
- Forwarding: It means that a message goes down in the network tree until it reaches its destination sub-network. This routing type is also based on the vID to route packets.
- Parsing: It is the last step for a message transmission. This type is used when a message reaches the destination sub-network, and it is going to be delivered to the destination node, using from now on the node address to route.

**Figure 10:**
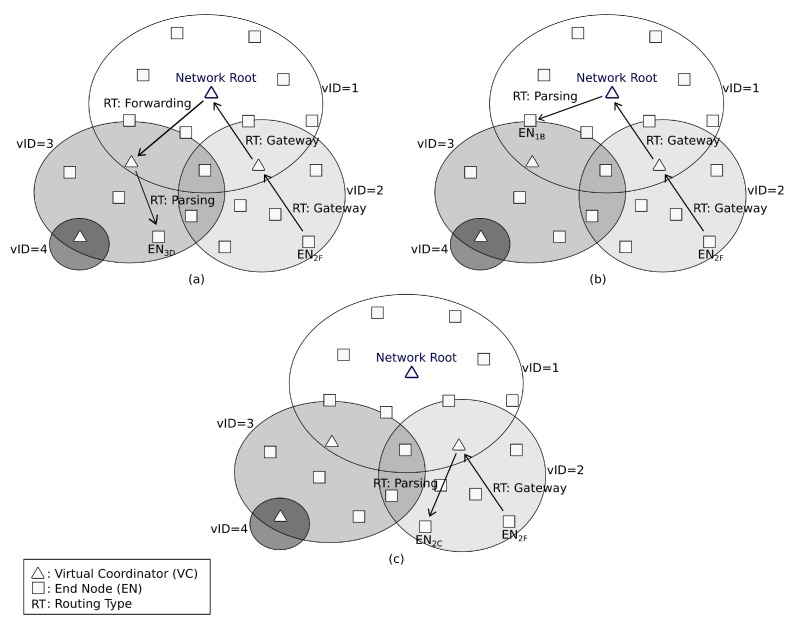
Example of different routing types in *DARAL*: (**a,b**) inter-cluster communication; and (**c**) intra-cluster communication.

Figure 10 shows examples of these kinds of messages graphically. The nodes represented as a triangle show the VCs, which manage a virtual sub-network. Cases Figure 10a,b show a communication between different clusters. In both cases, the node $EN_{2F}$ generates a packet with destination $EN_{3D}$ in Figure 10a and $EN_{1B}$ in Figure 10b. As long as $EN_{2F}$ is an EN without routing tables, it needs to send the message with the routing type *Gateway* to its VC. For this example, the VC from the sub-network 2 does not know the destination node, so it also routes the message to its VC (in this case, the *Network Root*) using also the routing type *Gateway*. As the *Network Root* knows the complete routing tree, it can find a route to forward the packet using the routing type *Forwarding* Figure 10a, or find the destination node using the routing type *Parsing* Figure 10b.

Another example is shown in Figure 10c, and it shows an intra-cluster communication. As long as a node sends a message to another node in the same sub-network, the VC can route it directly without external intervention i.e., the $EN_{2F}$ sends a message to $EN_{2C}$. The message goes firstly to its VC, which finds the node in the sub-network. Then, the VC sends the message to $EN_{2C}$ with the routing type *Parsing*.

## 4. Simulation

This section presents the simulation environment and methodology used to evaluate the behaviour of *DARAL* in Smart City scenarios. The simulations have been performed with *OMNeT++* [32], a well-known C++ discrete event simulator in the research community. As OMNeT++ is not focused on wireless networks, it is necessary the use of a framework, as inetmanet [33]. *DARAL* was implemented for *OMNeT++* and it is publicly available in [34]. The network is configured following the Smart City scenario proposal taken from the literature review, using static nodes randomly distributed. As *DARAL* is an *IEEE 802.15.4*-based routing algorithm, the communication band used is the ISM band of 2.4 GHz using the beaconless mode jointly with the *IEEE802154RadioModel* of inetmanet. Several experiments were carried out tuning the different parameters $T_{link}$, $T_{alive}$, $T_{down}$, $T_{reconnect}$, $L_{nodes}$, $TH_{baselevel}$ and $TH_{role}$. During this tune phase, $T_{reconnect}$ showed a strong influence in the performance of the protocol, affecting both the convergence time and the number of messages sent during the set-up phase. Due to this effect, we have chosen a low value for $T_{reconnect}$, prioritizing a low convergence time and by extension a low energy consumption over the number of messages sent. Table 1 shows a summary of the most relevant parameters for physical, MAC and network layers jointly with their values. For a better understanding, Appendix B presents a deep explanation of each parameter of *DARAL*.

**Figure 11:**
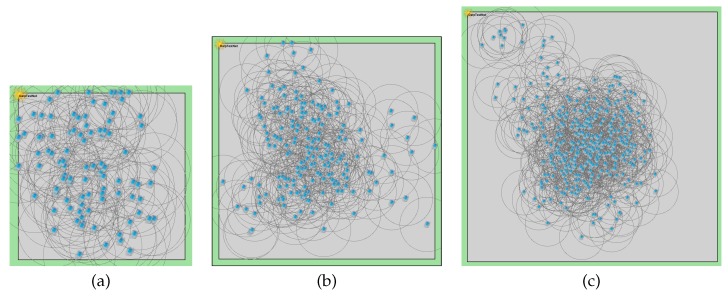
Samples of a small (**a**), medium (**b**), and large (**c**) scenarios.

Considering the different possibilities in a Smart City scenario, it is necessary to define different workloads in terms of traffic. There are different possibilities for that, and all of them involve the area size and the number of nodes, so the modification of these parameters makes possible to work with different network densities using the network degree as reference. For this work, three different node degrees (ND) are considered: 5, 10 and 15. The smallest scenario is built in a 145×145 m and the largest in a 500×500 m area. The use of this wide area and node degree combinations allows the simulation from sparse networks to highly dense networks. As a result, the simulation covers several network densities and sizes, resulting in a vast results comparison. The different combinations between network size, grade and number of nodes are shown in Table 2. For each experiment configuration, 10 randomly generated scenarios with different random seeds were evaluated. Figure 11 shows a scenario overview, taking random scenarios from the simulations and showing the nodes and their radio range.

In order to be able to compare our results with those of *O-RPL*, we have used the configuration used by Kermajani et al. in [7], setting the redundancy constant (*K*) to two. This value is used due to the impossibility of simulating low-density scenarios with lower values of *K*. Thus, the use of the lower possible value is necessary due to the lack of a redundancy constant in *AODV* or *DARAL* and aiming to develop a fair comparison.

### 4.1. Metrics Overview

This section details how the different parameters analysed are measured, giving a better overview of the metrics used.

#### 4.1.1. Convergence Time

The convergence time is a key parameter in the present work, and it has been calculated differently for each routing protocol, granting a fair comparison between different algorithms such as *AODV* and *O-RPL*.

For *AODV*, the convergence time for each node is calculated independently as the elapsed time since a random node sends a Route Request (RREQ) message to an objective node until a Route Reply (RREP) message is received by the sender. The average convergence time of the whole network is calculated after all the nodes in the network have calculated their own convergence times, as the average of the individual convergence times of all the nodes in the network.

For *O-RPL*, it has been used the mechanism implemented by Kermajani et al. [7], where the convergence time of a network is measured as the time elapsed, since a new *DODAG* root is set until all the nodes have joined that *DODAG*. To obtain an average measure, 10 different routes have been calculated, changing the *DODAG* root for each one. Then, the average convergence time has been estimated as the mean of the convergence times obtained in these 10 initializations of the network.

Finally, for *DARAL*, the convergence time of each node is calculated as the elapsed time since the node is started until its status changes to *CONNECTED* or *AWAITING*, as both statuses allow a node to send and receive data messages. Then, the network convergence time is obtained as the average of all the node convergence times in the network.

#### 4.1.2. Number of Messages Sent during the Set-up Phase

The calculation of the number of messages sent during the set-up phase also depends on each routing algorithm.

For *AODV*, the number of messages for each node is calculated as the sum of all the messages sent for each *RREQ* message until the *RREP* message is received. The final number of messages of the whole network is obtained as the mean of the number of messages sent by all the nodes.

For *O-RPL*, the calculation is carried out by the *DODAG root*, counting the number of *DIO* messages sent, but discarding either *DIS* and *DAO* messages. As 10 different initializations with different *DODAG* roots are simulated for each network, the final number of messages is obtained as the mean number of messages of these 10 simulations.

For *DARAL*, all the control messages sent by each node (*ASSOCIATION_REQ* and *ASSOCIATION_REP*) are counted, since it is started until it changes its state to either *CONNECTED* or *AWAITING*. Then, the final number of messages for the network is calculated as the mean number of messages sent by all the nodes.

#### 4.1.3. Energy Consumption

The study of the energy consumption is focused on the set-up or connection phase, exactly in the interval until the node is connected. The energy configuration, battery capacity and consumption parameters have been identically set, so the result in miliwatts per second mWs is directly measurable and comparable. Each algorithm has either a variable that measures the energy consumed or a variable that stores the remaining energy in the battery. In that case, using the remaining capacity and knowing the full capacity of the battery makes it possible to estimate the energy used. Once the connection phase is finished, the energy consumption of the whole network is estimated as the mean of the energy consumption for all the nodes in the net.

### 4.2. Set-up Phase Convergence Time

**Figure 12:**
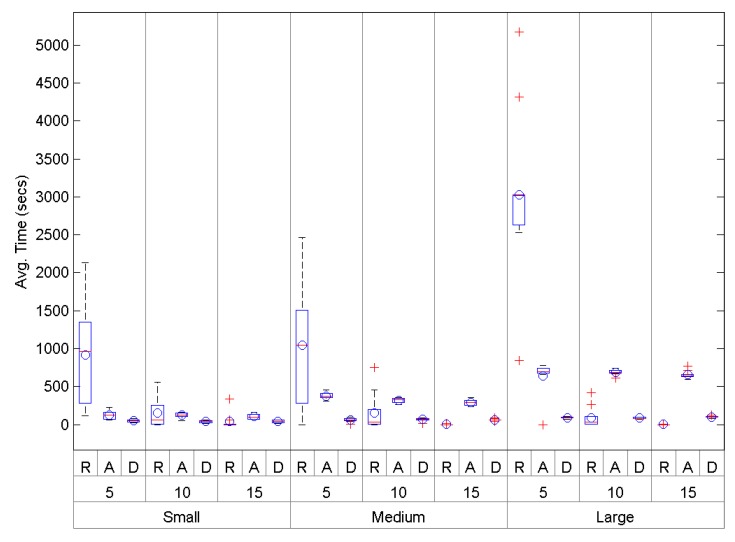
Convergence time results for small-, medium- and large-size scenarios.

Figure 12 shows a global comparison of the average network convergence time (or set-up time) in small-, medium- and large-size scenarios for *AODV* (A), *O-RPL* (R) and *DARAL* (D). As long as the area size is directly related to the node degree, it is not considered in the graphical representation. As average convergence times are shown, smaller values mean better performances, specifically, Figure 12. Small and Table 3 show a small-size scenario with 100 nodes and the differences for each node density. For small-size scenarios with ND lower or equal to 10, *DARAL* shows a better performance than *AODV* and *O-RPL*. Nevertheless, *O-RPL* performance is slightly better for scenarios with a high density, as can be seen in the case of ND=15, even though the results of *O-RPL* are not significant due to their standard deviation. Figure 12. Medium shows a medium-size scenario with 200 nodes, where the results are similar to the small-size scenario. The performance of *O-RPL* in the ND=15 case is so low that it cannot be observed in the graphical representation, although it can be seen in Table 3. Figure 12. Large shows a large-size scenario with 400 nodes, varying also in terms of density. As in the other scenarios, the different values for each protocol can be seen in Table 3. *DARAL* is repeated as the best solution for ND=5 and ND=10. For ND=15, there is no doubt about *O-RPL*, showing an extremely good performance in terms of convergence time and exceeding *DARAL* and *AODV*. Globally, *DARAL* shows the best performance for low- and medium-densities. Under these conditions and depending on the scenario, *AODV* also improves *O-RPL*, but under high-density conditions, *O-RPL* gives the best performance.

### 4.3. Number of Messages Sent during the Set-Up Phase

**Figure 13:**
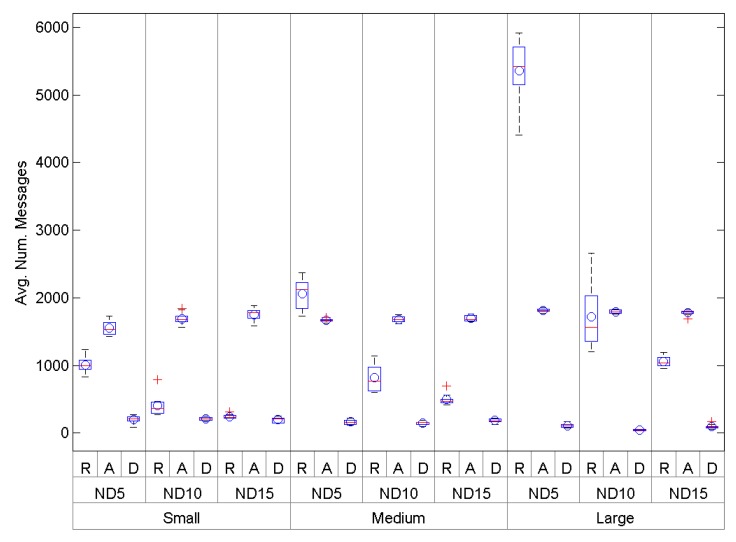
Average number of messages to set-up the network for small-, medium- and large-size scenarios considering *AODV*.

The number of messages involved in the network setting-up phase can be used as a good estimator of the protocol control overhead. Figure 13 shows a global comparison between *O-RPL* (R), *AODV* (A) and *DARAL* (D). Therefore, Table 4 also shows the results. As we can see, DARAL seems to be more efficient in terms of number of messages. The relationships between these differences has been statistically analysed. Every sample was independently analysed using a one-sample Kolmogorov–Smirnov test, resulting in a rejection of the data normality. Then, we proceeded to run a Kruskal–Wallis test, and the results showed that the differences between *O-RPL*, *AODV* and *DARAL* are statistically significative.

### 4.4. Energy Consumption during the Set-up Phase

**Figure 14:**
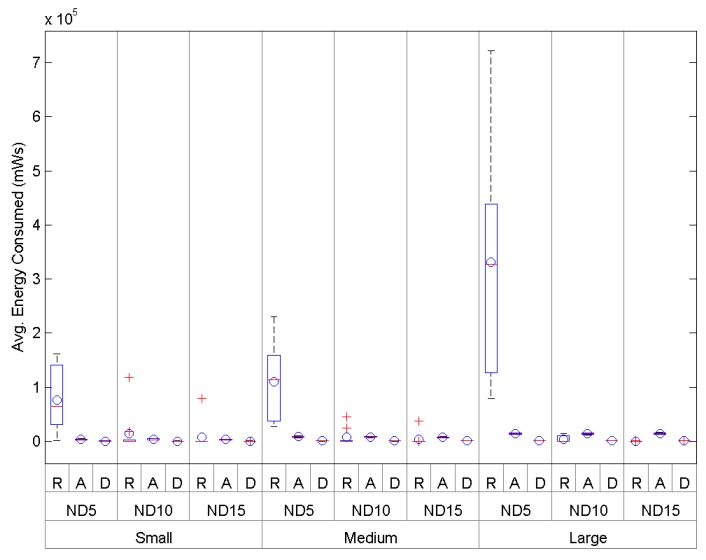
Comparison of the energy consumption in small-, medium- and large-size scenarios during the set-up phase.

In Figure 14, Small shows a high energy use of *O-RPL* (R) in small-size scenarios under low-density conditions, but with a major reduction under medium- and large-density conditions. *AODV* (A) shows an equilibrated performance, showing a lower performance than *DARAL* (D), which, on the other hand, presents the best results as Table 5 shows, requiring around 527–574 mWs depending on the conditions. *DARAL* also presents the lowest maximum and minimum, and a low dispersion, regardless of the scenario conditions.

Finally, in Figure 14, Large shows a low performance for *O-RPL* in large-size scenarios under low-density conditions. *AODV* also improves, but the performance is worse than *O-RPL* and *DARAL* under medium- and high-density conditions. *O-RPL* presents a notable improvement between low-, medium- and high-density conditions, even improving *DARAL* in this last case. *DARAL* shows again an equilibrated performance under different density conditions, using 850–896mWs, which is a tight range. The results from *O-RPL* are interesting, but according to the deviation values showed in Table 5, they are not significant enough, whereas *DARAL* results are much more robust.

In Figure 14, Medium shows the energy consumption comparison in medium-size scenarios, where *O-RPL* newly presents a low performance under low-density conditions. For the other density conditions analysed, *O-RPL* presents a slight improvement with respect to *AODV*. *DARAL* improves both algorithms, requiring 702–716 mWs, far less than *O-RPL*, which needs 3.78e+03 mWs, as Table 5 shows. In terms of lowest maximum and minimum and dispersion, *DARAL* presents the best results.

## 5. Conclusions

This paper presents a new routing algorithm focused on the architecture of Smart City, where the locality principle is exploited. We analysed three key parameters for the Smart City, the convergence time of a network, the number of messages necessary to connect a node to the network (control overhead) and the energy consumption during the set-up phase. We carried out intense simulations to analyse and validate the routing algorithm, and to compare it with other well-known algorithms like *AODV* and *RPL*.

After analyzing the results in detail, the use of *DARAL* reduces the convergence time in small and medium scenarios for different densities, as well as in large scenarios with low and medium densities. For large scenarios with high density, *O-RPL* produces the best results, but *DARAL* shows better results for different scenarios and under different conditions, being more tolerant and stable to variations in the node density conditions. For example, *DARAL* convergence time remains under 100, 67 and 50 s for large-, medium- and small-size scenarios, respectively. *O-RPL* shows a wider range between low and high densities, reaching a maximum of 3022.7 s and a minimum of 0.76 s. *AODV* is also consistent in terms of stability, but results in the worst performance of these experiments. *AODV* results seem also to be dependent on the number of nodes, scaling from 100–120 to 640–688 s for the different scenarios, and resulting in a better performance for small scenarios with high density, being anyway slightly worse than *DARAL*.

Another performance analysis of *DARAL*, which is also based on a comparison between routing algorithms, is the control overhead. The results of this analysis show that *AODV* and *O-RPL* need too many messages to form the network graph, requiring more than 1500 messages in some conditions. *DARAL* needs an average of around 100 messages for large-size scenarios, but being specially optimized for medium density conditions, whereas *O-RPL* uses a wide range, which goes from 1055 for large-size scenarios to more than 5300 messages for small-size scenarios. It is possible to see the significance of *DARAL* due to its low standard deviation, maintaining a good performance in terms of messages for the set-up phase, and being the better option for low- and medium-density scenarios.

The last analysis is related to the energy consumption, showing an overall better performance of *DARAL* also in terms of significance. *O-RPL* shows a high energy consumption under low-density conditions, but with a major improvement under high-density conditions, regardless of the scenario size. *AODV* shows a number of nodes-related consumption, showing also a certain stability with respect to the density conditions.

Additionally, the authors mention that *DARAL* performance is dependent on diverse parameters such as $TH_{baselevel}$, $TH_{role}$, $L_{nodes}$ or the different timers such as $T_{reconnect}$, which strongly determines the performance of the algorithm. These parameters open up a wide spectrum of configuration and the possibility to finely tune the algorithm using more complex methods.

## Figures and Tables

**Table 1:** Most relevant configuration parameters for the simulation.

Parameter	Value
Carrier Frequency (GHz)	2.4
Carrier Sense Sensitivity (dBm)	–85
Transmit Power (mW)	1.0
$T_{link}$ (s)	1.0
$T_{alive}$ (s)	600.0
$T_{down}$ (s)	5.0
$T_{reconnect}$ (s)	2.0
$T_{ack}$ (s)	1.5
$L_{nodes}$	50
$TH_{baselevel}$	45
$TH_{role}$	80
Payload size (Bytes)	70
$MAX_{SimTime}$ (s)	3600

**Table 2:** Different simulation scenarioes.

Network Size	Number of Nodes	Area Size	Node Degree
Small	100	250×250 m	5
Small	100	175×175 m	10
Small	100	145×145 m	15
Medium	200	350×350 m	5
Medium	200	250×250 m	10
Medium	200	200×200 m	15
Large	400	500×500 m	5
Large	400	350×350 m	10
Large	400	290×290 m	15

**Table 3:** Average convergence time (seconds (secs)) in small-, medium- and large-size scenarios.

ND	Algorithm	Avg. ± dev Small-	Avg. ± dev Medium-	Avg. ± dev Large-Scenario
5	O-RPL	916.05 ± 982.93	1163.8 ± 737.02	3022.7 ± 2209.2
5	AODV	122.59 ± 55.53	377.08 ± 45.73	640.17 ± 227.99
5	DARAL	49.05 ± 17.61	60.51 ± 23.39	90.27 ± 11.18
10	O-RPL	154.67 ± 295.97	156.11 ± 253.97	91.28 ± 196.67
10	AODV	121.7 ± 33.9	317.95 ± 36.15	688.87 ± 35.46
10	DARAL	38.91 ± 15.81	66.68 ± 20.86	84.95 ± 9.41
15	O-RPL	40.6 ± 151.51	1.85 ± 5.25	0.76 ± 0.99
15	AODV	105.22 ± 33.28	292.41 ± 38.45	655.26 ± 49.85
15	DARAL	40.74 ± 18.59	61.02 ± 13.02	98.43 ± 11.98

**Table 4:** Average number of messages sent during the set-up phase in small-size scenarios.

ND	Algorithm	Small-	Avg. ± dev Medium-	Avg. ± dev Large-Scenario
5	O-RPL	1010 ± 120.48	2056.1 ± 217.99	5343.2 ± 472.64
5	AODV	1555.27 ± 9689.08	1674.66 ± 6413.87	1739.48 ± 10750.61
5	DARAL	201.42 ± 55.55	168.96 ± 37.30	112.59 ± 27.67
10	O-RPL	407.82 ± 161.11	815.12 ± 206.99	1722.5 ± 473.59
10	AODV	1674.25 ± 10418.46	1678.56 ± 9386.83	1694.73 ± 11821.05
10	DARAL	211.75 ± 32.03	149.90 ± 25.98	47.29 ± 9.7
15	O-RPL	245.85 ± 31.81	496.63 ± 85.01	1055.2 ± 81.77
15	AODV	1739.48 ± 9723.29	1793.78 ± 8606.69	1777.7 ± 14901.79
15	DARAL	201.39 ± 39.8	186.53 ± 29.27	96.04 ± 33.36

**Table 5:** Average energy consumption (mWs) in small-, medium- and large-size scenarios during the set-up phase.

ND	Algorithm	Avg. ± dev Small-	Avg. ± dev Medium-	Avg. ± dev Large-Scenario
5	O-RPL	7.62e+04 ± 5.99e+04	1.09e+05 ± 7.08e+04	3.32e+05 ± 2.11e+05
5	AODV	3.36e+03 ± 817.03	8.49e+03 ± 1.13e+03	1.43e+04 ± 955.44
5	DARAL	574.88 ± 61.14	702.17 ± 112.57	870.02 ± 81.90
10	O-RPL	1.39e+04 ± 3.69e+04	7.20e+03 ± 1.55e+04	4.14e+03 ± 6.11e+03
10	AODV	3.31e+03 ± 1.08e+03	7.88e+03 ± 907.87	1.44e+04 ± 1.09e+03
10	DARAL	555.51 ± 67.48	712.61 ± 88.69	850.43 ± 59.11
15	O-RPL	7.89e+03 ± 2.49e+04	3.78e+03 ± 1.18e+04	96.15 ± 190.41
15	AODV	3.29e+03 ± 677.98	7.25e+03 ± 901.97	1.42e+04 ± 1.04e+03
15	DARAL	527.38 ± 74.86	716.27 ± 62.52	896.59 ± 59.13

## Appendix A. DARAL Messages Type Appendix

This appendix describes the different control messages present in *DARAL*, which totals 15 different messages, used for connection, deletion and routing tasks. The following is a short description of these messages:

As it is well known, every layer in a network communication stack adds a control header to the packet. Table A1 shows the *DARAL* header right after the *IEEE 802.15.4* MAC header in a network layer packet. It totals 27 octets, distributed as: operation code, which identifies the different control messages; packet length, used to read the payload length; routing type, which indicates the type of routing; a hop limit, not used by the moment; checksum and message id, used to avoid errors and duplication; source and destination virtual network id, used to primarily address the messages; source and destination address used to address the messages in the last step.

Operation code defines the type of control message used by *DARAL*, which totals 15 different messages, used for connection, deletion and routing tasks. These messages are described below:

- ASSOCIATION_REQ: Each node sends this message as a broadcast, to indicate that it requests a connection.
- ASSOCIATION_REP: A VC answers the *ASSOCIATION_REQ* message with this one, indicating that it can accept the node.
- ASSOCIATION_REP_ACK: If a node configures itself as EN, it sends this message to its VC.
- ASSOCIATION_PAN_ID_REQ: If a node configures itself as VC, it sends this message to its VC (also known as father), requiring a new vID. This message contains the information that identifies the node.
- ASSOCIATION_PAN_ID_REQ_ACK: This message is the answer from the network root to the *ASSOCIATION_PAN_ID_REQ*, and it contains the updated information (the new vID) for the node.
- ASSOCIATION_PAN_ID_ASSIGN: The VC (or father) assigns the new vID to the new VC.
- ASSOCIATION_PAN_ID_ASSIGN_ACK: This message is an answer to the *ASSOCIATION_PAN_ID_ASSIGN* message, and it confirms that the new sub-network is currently operative.
- ASSOCIATION_INFORM: A certain VC sends this message to the network root when a new node (VC or EN) is connected in its sub-network.
- ASSOCIATION_INFORM_ACK: It is the network root acknowledgement to an *ASSOCIATION_INFORM* message.
- KEEP_ALIVE_REQ: This message is the request of a VC for every node in its sub-network, in order to test the nodes’ connectivity
- KEEP_ALIVE_REQ_ACK: A specific answer for a *KEEP_ALIVE_REQ* message.
- PURGE_REQ: A VC sends this message to the network root when detects that a node in its sub-network is down.
- PURGE_REQ_ACK: This message is generated by the network root as response for a *PURGE_REQ* and it informs each node, until the destination, that a certain node is not available anymore.
- DATA: Sends a data frame, it can contain other protocol frames (e.g., 6LoWPAN-IPv6).
- DATA_ACK: It confirms the reception of a *DATA* frame.

All of these messages are used by the protocol to carry out different actions.

**Table A1:** *DARAL* control header description.

Offset	Field	Size (Octets)
0	Operation Code	1
1	Packet Length	1
2	Routing Type	1
3	Hop Limit	1
4	Checksum	2
6	Message Identificator	1
7	Source Virtual Network Identificator	2
9	Destination Virtual Network Identificator	2
11	Source Address	8
19	Destination Address	8

## Appendix B. DARAL Parameter Appendix

This appendix describes the different parameters of *DARAL*. A routing algorithm with an elevated number of parameters could be difficult to handle, but also allows a better tuning for system, compared to other fixed algorithms.

As every network layer algorithm and protocol, DARAL needs different timers regulating its functionality. Those timers regulate the answer of the algorithm against different events. The list of timers for DARAL is:

- $T_{link}$: The timer for link evaluation is fired when a node receives the first answer from a VC. $T_{link}$ represents the time that a certain node awaits to receive more answers from different VCs. Once this timer is expired, the connection process starts.
- $T_{alive}$: This timer fires the maintenance task of the network topology. Once it is expired, a certain VC tests its own sub-network, checking the connectivity with every node.
- $T_{down}$: This timer is used by a VC to protect its sub-network from node drops. When a certain node surpasses $T_{down}$ without responding messages from its VC, the VC proceeds to remove the node from the sub-network, informing to the topology about this drop.
- $T_{reconnect}$: This timer is used for two different tasks. First, it is used by a VC without vID, to resend the vID request to its VC. Secondly, it is used by non-connected nodes to restart the network stack, restarting also the connection process (sending a new broadcast asking for a valid link).
- $T_{ack}$: The timer for acknowledgement (ACK) measures the time that a certain message is valid. If an ACK is not received before the timer expiration, the packet should be resend. This kind of timer is typical in the network layer.

*DARAL* also makes use of two different thresholds to delimit the minimum link quality and the role played by a node:

- $TH_{baselevel}$: The base level threshold represents the minimum LQI that a link between nodes should have. If a link is not above this threshold, the node itself discards the connection and looks for another one.
- $TH_{role}$: The role threshold is used to determine the role of each node in the network. If LQI is above $TH_{role}$, a node connects to the network as EN, representing a good QoS and allowing that node to save energy adjusting its radio transceiver and reducing the interference with other nodes. If LQI is below $TH_{role}$, a node connects itself as VC, representing an extension for the coverage of the network.

Finally, *DARAL* makes use of another parameter ($L_{nodes}$) that balances the load of sub-networks:

- $L_{nodes}$: The node limit is a parameter that set the maximum number of nodes that a VC can handle. This limit balances the load between different sub-networks, creating areas with restricted traffic, due to the locality principle.

All of these parameters could be tuned by intelligent systems, opening a vast research line, in order to adapt them to diverse scenarios, not only for Smart Cities, but also for IoT.
